# Supplementary material for: Exploring the impact of drug decriminalization and legalization policies on mental health outcomes: A scoping review
Source: PLOS Ment Health. 2025 Oct 15;2(10):e0000358. doi: 10.1371/journal.pmen.0000358 (PMC12798171; doi:10.1371/journal.pmen.0000358)
Supplement: S1 Appendix — (DOCX) [file pmen.0000358.s001.docx]

| **#** | **Query** | **Results from 12 Dec 2023** |
| --- | --- | --- |
| 1 | (drug* or substance* or cannabis or heroin or marijuana or opioid* or narcotic* or psychedelic*).mp. | 13,523,631 |
| 2 | (de-criminali* or decriminali* or legali* or liberali* or commerciali* or depenali*).mp. | 32,199 |
| 3 | 1 and 2 [Drug decriminalization] | 13,768 |
| 4 | ((mental* or psychological) adj3 (health or disorder* or illness* or wellbeing or wellness or well-being)).mp. | 571,355 |
| 5 | (psychiatr* adj3 (symptom* or disorder* or illness* or visit* or hospital* or admission*)).mp. | 147,406 |
| 6 | amphetamine-related disorders/ or cocaine-related disorders/ or inhalant abuse/ or marijuana abuse/ or "marijuana use"/ or narcotic-related disorders/ or psychoses, substance-induced/ | 151,108 |
| 7 | Psychosis/ | 108,288 |
| 8 | (Psychotic or psychos*).mp. | 408,679 |
| 9 | or/4-8 [Mental health-related outcomes] | 1,049,470 |
| 10 | 3 and 9 | 2,613 |
| 11 | limit 10 to english language | 2,532 |
| 12 | limit 11 to yr="2001 -Current" | 2,503 |
