# Supplementary material for: Exploring the impact of drug decriminalization and legalization policies on mental health outcomes: A scoping review
Source: PLOS Ment Health. 2025 Oct 15;2(10):e0000358. doi: 10.1371/journal.pmen.0000358 (PMC12798171; doi:10.1371/journal.pmen.0000358)
Supplement: S2 Appendix — (DOCX) [file pmen.0000358.s002.docx]

**Data Extraction Tool**

**Title**

Title of paper / abstract / report that data are extracted from

**Lead author**

**Year of publication**

**Country in which the study conducted**

**Province(s)/state(s)**

**Type of policy change**

You can select multiple options

1. Medical legalization
2. Recreational legalization
3. Decriminalization
4. Commercialization
5. Other

**Class of substance(s)**

You can select multiple options. If "other" is selected, please specify.

1. Cannabis (Marijuana)
2. Opioids
3. Stimulants (Cocaine, Amphetamine))
4. Hallucinogens (LSD, Psilocybin)
5. MDMA (Ecstasy)
6. Other

**Date(s) of policy enactment**

**Methods**

**Aim of study**

**Study design**

If "other" is selected, please specify. For review papers, please specify the type of review in "other".

1. Randomised controlled trial
2. Quasi-experimental study (e.g., time-series)
3. Cohort study
4. Cross sectional study (e.g., repeated)
5. Case control study
6. Review paper
7. Qualitative research
8. Policy analysis
9. Other

**Start date**

**End date**

**Data sources**

**Target population**

**Sample size**

**Sample Characteristics**

Put N/A if information is not available. Please don't leave the cells empty.

|  | **Study group** | **Comparison group** | **Overall** |
| --- | --- | --- | --- |
| **Age** |  |  |  |
| **Sex/gender** |  |  |  |
| **Socioeconomic status** |  |  |  |
| **Education** |  |  |  |
| **Race/ethnicity** |  |  |  |
| **Rural/urban residence** |  |  |  |
| **History of drug use** |  |  |  |
| **History of mental illness** |  |  |  |
| **History of other illnesses** |  |  |  |
| **Other (specify)** |  |  |  |
| **Total** |  |  |  |

**Mental health outcome(s)**

Name the outcome and its indicator. Only extract outcomes that are related to mental health (e.g., psychosis, suicide, anxiety)

|  | **Outcome** | **Indicator/instrument** |
| --- | --- | --- |
| **Outcome 1** |  |  |
| **Outcome 2** |  |  |
| **Outcome 3** |  |  |
| **Outcome 4** |  |  |
| **Outcome 5** |  |  |
| **Outcome 6** |  |  |
| **Outcome 7** |  |  |
| **Outcome 8** |  |  |

**Statistical methods**

**Impact measure**

1. Prevalence difference
2. Mean difference
3. Median difference
4. OR/AOR
5. RR
6. Other

**Outcome table**

|  | **Effect size** | **Ratio** | **SD** | **CI** | **P value** |
| --- | --- | --- | --- | --- | --- |
| **Outcome 1** |  |  |  |  |  |
| **Outcome 2** |  |  |  |  |  |
| **Outcome 3** |  |  |  |  |  |
| **Outcome 4** |  |  |  |  |  |
| **Outcome 5** |  |  |  |  |  |
| **Outcome 6** |  |  |  |  |  |
| **Outcome 7** |  |  |  |  |  |
| **Outcome 8** |  |  |  |  |  |

**Key findings**

Any relevant finding that is not reported in a quantitative manner, including qualitative, meta-analysis, recommendation, etc.
