# Supplementary material for: Exploring the impact of drug decriminalization and legalization policies on mental health outcomes: A scoping review
Source: PLOS Ment Health. 2025 Oct 15;2(10):e0000358. doi: 10.1371/journal.pmen.0000358 (PMC12798171; doi:10.1371/journal.pmen.0000358)
Supplement: Attachment — Submitted filename: Rebuttal letter_PLOS.docx [file pmen.0000358.s005.docx]

May 30, 2025

Dear Prof. Cumming,
Academic Editor of PLOS Mental Health,

We would like to sincerely thank you and the reviewers for your time, effort, and thoughtful feedback on our manuscript. We acknowledge that the submitted version had several shortcomings, particularly regarding language clarity and the need to better address contextual factors influencing diverse outcomes. These limitations made cross-study comparisons more difficult.

With the benefit of your and the reviewers’ valuable insights, we have undertaken substantial revisions. We believe the revised manuscript now more clearly articulates our findings and addresses the concerns raised. We have also made additional refinements throughout the text to improve flow and readability.

Enclosed with this letter, please find our revised manuscript (PMEN-D-25-00024), entitled *“Exploring the Impact of Drug Decriminalization and Legalization Policies on Mental Health Outcomes: A Scoping Review.”* A detailed, point-by-point response to each reviewer’s comments is attached below.

We thank Reviewer 1 for their careful reading of our work and valuable suggestions, which helped enhance both the structure and the contextual framing of the paper.

We thank Reviewer 2 for their insightful and detailed feedback, which greatly contributed to strengthening the manuscript’s relevance, analysis, and discussion.

Once again, we are grateful to the editorial team and all the reviewers for their contributions. We hope our revisions adequately address all concerns and meet the expectations of *PLOS Mental Health*.

Sincerely,

On behalf of the authors,

Mana Mohebbian,

PhD Candidate,

University of British Columbia

**Journal Requirements:**

1. Figure 2: please (a) provide a direct link to the base layer of the map (i.e., the country or region border shape) and ensure this is also included in the figure legend; and (b) provide a link to the terms of use / license information for the base layer image or shapefile. We cannot publish proprietary or copyrighted maps (e.g. Google Maps, Mapquest) and the terms of use for your map base layer must be compatible with our CC-BY 4.0 license.
Note: if you created the map in a software program like R or ArcGIS, please locate and indicate the source of the basemap shapefile onto which data has been plotted.
If your map was obtained from a copyrighted source please amend the figure so that the base map used is from an openly available source. Alternatively, please provide explicit written permission from the copyright holder granting you the right to publish the material under our CC-BY 4.0 license.
Please note that the following CC BY licenses are compatible with PLOS license: CC BY 4.0, CC BY 2.0 and CC BY 3.0, meanwhile such licenses as CC BY-ND 3.0 and others are not compatible due to additional restrictions.
If you are unsure whether you can use a map or not, please do reach out and we will be able to help you. The following websites are good examples of where you can source open access or public domain maps:
* U.S. Geological Survey (USGS) - All maps are in the public domain. ([http://www.usgs.gov](http://www.usgs.gov/))
* PlaniGlobe - All maps are published under a Creative Commons license so please cite “PlaniGlobe, [http://www.planiglobe.com](http://www.planiglobe.com/), CC BY 2.0” in the image credit after the caption. (<http://www.planiglobe.com/?lang=enl>)
* Natural Earth - All maps are public domain. (<http://www.naturalearthdata.com/about/terms-of-use/>)

***Response:***

To generate the world map for Figure 2, we used the Natural Earth low-resolution dataset, which is bundled with GeoPandas in Python. The underlying shapefile comes from:

**Natural Earth, 1:110m Cultural Vectors (Admin 0 – Countries)**

<https://www.naturalearthdata.com/downloads/110m-cultural-vectors/>

Regarding the License, Natural Earth datasets are in the public domain at:

<https://www.naturalearthdata.com/about/terms-of-use/>

It is stated that *“No permission is needed to use Natural Earth. Crediting the authors is unnecessary.”* — which is compatible with CC-BY 4.0.

The following phrase has been added to the Fig 2 caption on page _ line _ to clarify the underlying shapefile and terms of use:

*“ Base layer source: Natural Earth, 1:110m Cultural Vectors (Admin 0 – Countries), available at https://www.naturalearthdata.com/downloads/110m-cultural-vectors/ (public domain; terms of use:* [*https://www.naturalearthdata.com/about/terms-of-use/*](https://www.naturalearthdata.com/about/terms-of-use/)*)).”*

**Additional Editor Comments (if provided):**

Please carefully review the important points made by the two expert reviewers. In particular attention should be paid to their comments around the language used, to ensure that you describe substances and substance use in a way that is contemporary and non-stigmatising. Also, the bi-directionality of the relationship between substance use and mental health issues should be acknowledged in greater detail to reflect the sizeable evidence base showing that causation can flow in either direction.

In your analysis and discussion around the “direction of evidence” starting on p24, there appears to be some important context missing. One of the issues is that while different jurisdictions may implement policies aimed at decriminalizing or legalizing the use of substances that were previously illegal to use, the circumstances in which they do this can differ greatly. For example, in Portugal, alongside decriminalisation of the use of previously illicit substances, there was also a substantial increase in investment in health interventions so that people identified as having a problem with substance use could be referred into treatment, and away from the justice system. The evidence suggests that the investment in health resources was largely sufficient to meet demand, with notable decreases in the rates of drug-related issues such as mental illness, overdoses and blood borne viruses as a result. This contrasts with the experience in Oregan which also decriminalized formerly illicit substance use. While additional funding was allocated to health services to help treat substance-related problems, the level of investment in these services has largely been viewed as inadequate to meet demand. Considering these contextual factors is important when interpreting the literature from different jurisdictions and assessing whether a change in drug policy also included sufficient investment in health resources to handle increased demand created by diverting people away from the justice system, and into health services. This will have an important impact on whether the rates of substance-related mental health issues changed, and in what direction they moved. Not giving consideration to contextual factors such as these will limit how well your review informs future policy and practice in the area.

***Response:***

We fully agree that a context-sensitive lens was missing from our analysis, and we recognize that understanding the context in which drug policy changes are implemented is critical to accurately interpreting their impact—particularly with respect to mental health outcomes.

In response, we have revised the Discussion section to more clearly acknowledge and explore the role of contextual factors. As you rightly point out, examples like Portugal and Oregon highlight how similar policy changes (e.g., decriminalization) can produce very different outcomes depending on the supporting health and social systems.

While we have not analyzed these contextual factors in our scoping review, we now discuss that the overall of impact observed in individual studies cannot be fully understood without considering how well and in what setting these policies have been emplemented. We also added that caution should be exercised when comparing studies across countries or drawing conclusions from aggregated findings.

Please find the revised paragraph on page 34 line 792:

*“Comparing the mental-health impact of liberal drug policies across jurisdictions is inherently challenging because outcomes depend not only on the policy text but also on how—and how well—the policy is implemented. Portugal’s landmark 2001 reform, for instance, paired nationwide decriminalization with substantial investment in treatment and harm-reduction programmes through its “Commissions for the Dissuasion of Drug Addiction,” a package linked to declines in drug-related harms and psychiatric sequelae [89]. British Columbia has opted for a slower, pilot-based approach within a tighter legislative framework [90, 91], while Oregon’s 2021 decriminalisation earmarked additional health funding that some observers view as inadequate, potentially blunting mental-health benefits (Oregon Health Authority & Oversight and Accountability Council 2023). These examples underscore that “decriminalization” is not a uniform intervention: local enforcement practices, health-system capacity, and complementary regulations can strongly influence psychiatric outcomes. Our review did not analyze these contextual factors in depth; therefore, future studies should explicitly account for such variables—linking mental-health trends to strategic planning, infrastructure investment, and pre-policy conditions—to provide a more accurate appraisal of overall policy impact.”*

**Response to Reviewer #1 Comments:**

This paper reports on a scoping review conducted to explore any literature about the impact of drug policies (namely decriminalisation, legalisation, and commercialisation) on mental health outcomes. The paper specifically seeks to expand previous reviews of cannabis policies and mental health outcomes to include drugs other than cannabis. This paper also includes an extended timeframe and broader range of cannabis policies.

A huge strength of this paper is the reporting and synthesising of both reviews and original papers. This helps to interpret the findings in relation to previous reviews, identify areas with less clarity, and areas where the evidence base is stronger. It is also an incredibly useful finding that there does not appear to be any research on drugs other than cannabis in relation to MH outcomes.

***Comment:***

My main feedback for this paper is that it could be strengthened by focusing less on the direction of evidence and more on the study designs, policy frameworks, and outcomes examined. Given this is a scoping review, I feel at times that the direction of evidence requires further contextualising, which is difficult due to the wide range of methods, outcomes, and policy interventions studied. At present, I believe some of the studies are being misrepresented as statements about direction of evidence are being made. An example is Lira (45) – stricter cannabis policies associated with less cannabis involvement in suicides/other deaths is summarised as ‘increase’, but I’m unclear about the assumption here (as it does not state whether suicides increased or decreased, only whether cannabis was involved). It may be that more people use cannabis, but not whether the increase in use is also associated with increases in suicide.

***Response:***

Thank you for this thoughtful observation. In response, we have refined our presentation of findings and changed the terminology from “direction of evidence” to “overall policy impact” to improve clarity and avoid implying unwarranted precision. Our intent is to provide a descriptive summary of each individual study’s findings, rather than to draw broad or definitive conclusions.

We have also aimed to limit our discussion of the “overall impact” and instead shift the focus to the identified gaps. We also clarified in the Discussion section that various contextual factors—such as health system capacity, enforcement practices, and policy implementation differences—can significantly influence outcomes and may impede direct comparisons or causal inferences. Therefore, this overall impact classification is presented as a high-level interpretive tool, not as a substitute for nuanced, context-aware analysis. In addition, to address your comment regarding the ambiguity of the findings in the paper by Lira et al., we have rephrased the sentence to provide a clearer interpretation.

Please find the revised text in table 1 on page 18 :

*“Lira et al* – *More liberal state-level cannabis policies were linked to higher odds of cannabis involvement in suicides and unintentional injury deaths.”*

***Comment:***

I’ve included a short description from another paper about scoping reviews which may assist:
“Scoping reviews synthesize evidence and assess the scope of literature on a topic. The value of adopting a scoping review methodology lies in its broader approach to mapping literature and addressing research questions than other systematic reviews. Unlike systematic reviews, the aim of a scoping review is to map the key concepts that underpin a research area (Arksey & O’Malley, Citation2005). Scoping reviews are commonly conducted to summarize and disseminate research findings, identify research gaps, and make recommendations for future research. But they may also be carried out to determine the way the research has been conducted (Peters et al., Citation2015); that is, to provide useful insight into the nature of a key research concept, to clarify working definitions, and identify conceptual boundaries of a topic or field (Peters et al., Citation2015, Citation2021).”
<https://doi.org/10.1080/26895269.2024.2447785>

My suggestion is to revise to identify areas for further research and more closely examine the methods used in existing research. This might help inform evaluation of the direction of the evidence. I have some more detailed feedback which I’ve left below.

***Response:***

Thank you for sharing this helpful description and for directing us to the relevant reference. In response, we have revised the manuscript to more clearly articulate these aims. Specifically, we have:

- Expanded the Results section to include a more detailed description of the study designs and methodologies used in the reviewed papers.

Please find the added text on page 28 line 599 :

*“There were two qualitative studies in which data were collected through semi-structured interviews: one with healthcare providers in a psychiatric hospital setting [71], and the other with individuals who had a prescription for medical cannabis and their carers [40].”*

Please find the added text on page 28 line 606 :

*“The majority of studies (n=24) compared pre- and post-legalization trends, primarily using quasi-experimental (e.g., interrupted time series) and longitudinal designs. Fifteen studies compared states or regions that adopted the policy with those that did not, either alongside a pre-post approach or as a standalone comparison. Two studies constructed synthetic control groups to represent counterfactual scenarios in which the policy had not been implemented [39,45]. Additionally, two studies used conditions or emergency department visits unrelated to cannabis as comparison outcomes [42,51]”*

- Added a paragraph to the Discussion highlighting key gaps in the current literature and offering targeted recommendations to guide future research in this area.

Please find the added paragraph on page 36 line 864 :

*“To generate evidence that is both generalizable and policy-relevant, future studies must move beyond basic outcome assessments and adopt designs that account for broader policy and social contexts. This includes pre-policy conditions, the scale of harm reduction and public health efforts, implementation funding, and the availability of mental health and addiction services. Furthermore, studies should incorporate longer follow-up periods to capture both the immediate and long-term effects of policy changes, as some mental health outcomes may emerge or evolve well after the initial policy implementation.*

*Importantly, future research should also address the clear gap in evaluations of policy reforms targeting substances other than cannabis. Qualitative and mixed-methods approaches—incorporating the lived experiences of people who use drugs, community members, service providers, and policymakers—can reveal unintended consequences, community dynamics, and contextual factors shaping policy impact. By integrating these perspectives, research can better explain cross-jurisdictional variation and support the development of more nuanced, equitable, and effective drug policies.”*

***Comment:***

**-The section about relationship between substance use and mental health (introduction, para 4) cites a lot of research about substance use disorders and mental health outcomes. There might be a conflation here about substance use and substance use disorders. Many people use substances (including legal and illegal) and do not develop substance use disorders. The relationship between substance use disorders and other mental health disorders is quite different to the relationship between substance use and mental health outcomes. I would recommend clarifying this and possibly differentiating the research to be more specific. This will also avoid unnecessary stigma towards people who use drugs. Also, it may be worth noting that legal substances, such as alcohol, also are linked to MH outcomes.

***Response:***

We have revised the relevant section in the introduction to clearly distinguish between recreational substance use and substance use disorders (SUDs). We now clarify that the relationship between substance use and mental health is bidirectional, with mental health complications more commonly observed and studied among individuals with SUDs (co-occurring disorders). To help reduce stigma toward people who use drugs, we have emphasized that these mental health outcomes are influenced by multiple factors—including patterns of use (e.g., chronic or dependent use), substance potency, and frequency—rather than use alone and are usually observed with problematic use. Additionally, we have added a sentence noting that legal substances, such as alcohol, are also associated with mental health issues.

Please find the revised paragraphs on page 5 line 154 :

*“Beyond legal and economic effects, liberal drug policies may have profound implications for public mental health. Substance use and mental health share a complex, bidirectional relationship: each can precipitate or aggravate the other [17]. Although the primary mental health consequence of problematic drug use is the development of substance use disorders (SUDs), drug exposure can also induce a spectrum of other psychiatric symptoms, including depression, anxiety, and psychosis [18]. Even occasional or nondependent consumption may provoke mental health issues [19], yet these co-occurring complications are particularly prevalent among individuals with SUDs [20].*

*The nature and severity of these mental health effects depend on the type of substance, the pattern of use, and individual vulnerability. For example, early and heavy cannabis use in adolescence can disrupt neural maturation and heighten the risk of psychosis, depression, and suicidal ideation [21]. Problematic use of opioids—whether prescribed or illicit—are linked to depression, anxiety, and suicidality, especially during withdrawal phases [22,23]. Stimulants such as cocaine and amphetamines may trigger acute paranoia and psychotic episodes, with chronic use often resulting in persistent cognitive deficits [24,25]. Harmful use of legally available substances like alcohol use disorder can also elevate the risks of depressive and suicide [26]. These overlapping conditions not only complicate diagnosis and treatment but also point to shared biological and psychosocial vulnerabilities.”*

***Comment:***

**-The previous review by Fortier could be better discussed, in both the introduction and discussion. Things I would be interested to know are the timeframe they used (you note yours is a longer timeframe), their findings, how many studies were included, which outcomes they focused on, and which policy interventions. Also, in discussion you could note how many of your papers were covered in the Fortier paper, and how your findings compare/contrast.

***Response:***

We have revised both the Introduction and Discussion sections to provide a clearer comparison between our review and the previous review by Fortier et al. Specifically:

- In the last two paragraphs of the Introduction, we now include more details about the Fortier review, including its timeframe, number of included studies, and the types of policy interventions assessed. We also highlight the broader timeframe of our review, our inclusion of a wider range of mental health outcomes, and policy types not addressed in the earlier paper.

Please find the revised paragraph on page 6 line 198 :

*“. A recent scoping review by Fortier et al. investigated the mental‑health impacts of cannabis legalization [27], identifying twenty‑eight original studies and reports from January 1, 2012, to April 30, 2023. This paper has outlined the limited research available and highlighted the largely inconclusive findings, however, its focus was specifically on cannabis policies.*

*The present review expands the scope to include a broader range of drug policies—such as the decriminalization of psychoactive substances beyond cannabis, alcohol, tobacco, or prescribed medications—and extends the analysis period back to January 2001. We also incorporate review papers alongside original studies to map the entire body of existing literature. This approach offers a more comprehensive, up‑to‑date overview of current knowledge.”*

- In the Discussion, we expand on how our review builds upon Fortier et al., noting both areas of convergence and divergence in findings.

Please find the relevant paragraph on page 33 line 751 :

*“Our findings regarding the most frequently studied mental health outcomes are congruent with those reported by Fortier et al, with psychosis, suicide, and depression consistently identified as the most examined conditions in the context of liberal drug policies[27].”*

Please find the relevant paragraph on page 34 line 782 :

*“This finding was similar to a previous scoping review by Fortier that reported original studies and reports exclusively from Canada and the United States, with no articles originating from other countries with cannabis legalization[27].”*

Please find the revised paragraph on page 36 line 857 :

*“Despite its limitations, this review offers a broader perspective than Fortier et al. [27] by synthesizing findings from both original and review papers to locate all relevant publications. With extended scope and timeframe our review provides a more comprehensive overview of the current evidence, and highlights significant gaps in knowledge regarding study design, setting, and time.”*

***Comment:***

**-Line 138 – ‘occurrence of secondary mental health disorders, such as depression and anxiety, that are induced or triggered by drug use’. This might benefit from further clarification. Does this mean only MH disorders which are triggered by drug use? Are any excluded? And does secondary mean, in addition to substance use disorders? Throughout, I think this paper could benefit from greater differentiation between substance use and substance use disorders.

***Response:***

We agree that the term was unclear and have replaced it with more specific phrase “non-SUD mental disorders that may develop following exposure to psychoactive substances, regardless of whether the individual meets criteria for a substance use disorder.”

Additionally, we have revised the methods section to explicitly state that while SUDs are classified as mental health disorders, they were not included as outcomes of interest in this review. This decision was made to focus specifically on substance-related mental health outcomes rather than substance use disorder itself (such as depression, anxiety, psychosis) that may arise following drug use, whether dependent or non-dependent.

Please find the revised paragraph on page 9 line 283 :

*“The primary outcome of interest was the onset of non-SUD mental health complications—such as depression or anxiety disorders—that may develop following exposure to psychoactive substances, regardless of whether the individual meets criteria for a substance use disorder. This includes both acute mental health crises triggered by substance use (e.g., psychotic episodes during intoxication) and longer-lasting or recurrent disorders that may persist beyond the immediate effects of the substance [18,33]. We included studies reporting population-level measures derived from national statistics, administrative health records, or large cohort studies. Both large, population-based datasets and smaller clinical or community samples were accepted, provided they met our diagnostic or measurement criteria. Studies were included if they employed clinical diagnoses (based on DSM or ICD criteria), validated diagnostic interviews, prescription-fill records for major psychiatric medications, or standardized self-report scales to ascertain mental health outcomes. We excluded any studies that:*

*•	Reported solely on patterns of drug consumption (e.g., frequency, quantity) or on problematic use indicators (e.g., dependence, abuse) or all-cause drug-related emergency department visits (e.g., overdose rates, drug-related deaths, intoxication, or withdrawal) without measuring psychiatric symptoms;*

*•	Focused exclusively on substance use disorders (SUDs). While SUDs are recognized substance-related mental conditions, they were excluded to allow a focused examination of non-SUD substance-related mental health outcomes—such as depression, anxiety, or psychosis;*

*•	Investigated physical health outcomes, civic and social impacts, motor vehicle accidents, drug-related arrests, law enforcement activity, or any aspect of drug trafficking, manufacturing, or cultivation;*

*•	Addressed mental disorders associated exclusively with prescribed medications, alcohol, tobacco, or caffeine. Prescribed cannabis and psychedelics were treated as distinct exposures, as their medical legalization falls within the scope of this review. Here “prescribed medications” refers specifically to substances commonly used without policy-specific regulation (e.g., benzodiazepines).”*

***Comment:***

-Outcome section could benefit from examples. Eg. Population prevalence of MH disorders, any particular MH disorders examined… other measures? Only population/national studies or also smaller samples?

***Response:***

We have substantially expanded the Outcomes section to include concrete examples and to clarify the range of measure and studies included.

Please find the added paragraph on page 9 line 288 :

*“We included studies reporting population-level measures derived from national statistics, administrative health records, or large cohort studies. Both large, population-based datasets and smaller clinical or community samples were accepted, provided they met our diagnostic or measurement criteria. Studies were included if they employed clinical diagnoses (based on DSM or ICD criteria), validated diagnostic interviews, prescription-fill records for major psychiatric medications, or standardized self-report scales to ascertain mental health outcomes.”*

***Comment:***

**-Policy framework – I couldn’t find a definition for this. This is a part of your analysis, and some work is involved in deciding how to code the papers. This would be valuable to include, how you decided upon these categories and a simple description. Also, did any papers look at decriminalisation/other approaches other than legalisation? This would be really interesting to note as an area missing.

***Response:***

We have now added a clear definition of our “policy framework,” described how defined categories.

This is the revised paragraph on page 8 line 268:

*“Our initial aim was to assess the effects of liberal policies targeting non-cannabis psychoactive substances (e.g., opioids, stimulants) at national or jurisdictional level. Here, the “policy framework” encompasses three main regulatory approaches:*

*•	Decriminalization: removal of criminal penalties for possession or personal use, typically replacing them with administrative fines or diversion to treatment [30].*

*•	Legalization: establishment of a lawful pathway for production, distribution, and consumption under government oversight [31].*

*•	Commercialization: introduction of a regulated market in which private entities can produce, market, and sell substances, often alongside taxation and advertising controls [32].*

*Because our targeted search for studies on substances other than cannabis yielded no eligible papers, we broadened our scope to include evaluations of cannabis policies. Consequently, any study examining the impact of decriminalization, legalization, commercialization, or similar regulatory changes was considered eligible, regardless of substance class.”*

We have also highlighted in the results that no study examined “decriminalization” policy.

This is the revised paragraph on page 27 line 572 :

*“Notably, none of the original studies or review papers directly examined decriminalization as a distinct drug policy framework.”*

***Comment:***

-Sample sizes for each of the studies might be useful, as I found it difficult to know what size population was included. Eg Some of the qualitative papers I imagine were small sample sizes, but this isn’t clear.

***Response:***

We agree that reporting sample sizes improves transparency and interpretability. We have added a new column to report sample sizes for all original studies in the Table 1, where available.

Please see the revised table 1 on page 14 line 399.

***Comment:***

-I would recommend separating the qualitative studies, as I don’t know if they are best summarised with the quantitative studies. The language around ‘increases’, relationships, and influence throughout suggests largely quantitative analyses. I think being clear about the study design would help interpretation

***Response:***

We agree that the inclusion of qualitative studies alongside quantitative ones requires careful handling to avoid misinterpretation. While we chose not to separate the qualitative studies into a distinct category—as all original studies were coded and analyzed using the same framework—we revised the terminology used to describe findings throughout the manuscript and replace the term “direction of evidence” with “overall impact”. With this revision, instead of terms like *increase* or *decrease*, which imply quantitative measurement, we now use more inclusive language such as *positive*, *negative*, *mixed*, or *no impact* to better reflect both qualitative insights and quantitative trends.

Please see the revised Table 1 on page 14 line 399 and Table 2 on page 22 line 490.

***Comment:***

**-Some of the study outcomes might benefit from better explication. Eg Mental health medications is influenced by many other factors, and a correlation study with legalisation is problematic, as there are many state differences which may impact this.

***Response:***

We have revised the manuscript to provide greater detail and clarity regarding the mental health outcomes assessed across studies.

We fully acknowledge that patterns in psychiatric medication prescriptions can be influenced by a range of external factors. However, we chose to retain the one study that used changes in prescription fills as an outcome, as this measure—despite its limitations—can serve as a proxy indicator of trends in population-level mental symptoms. To address your concern, we have clarified in the results section that this indicator may not precisely reflect actual changes in mental health status.

Please find the revised paragraph on page 9 line 291 :

*“Studies were included if they employed clinical diagnoses (based on DSM or ICD criteria), validated diagnostic interviews, prescription-fill records for major psychiatric medications, or standardized self-report scales to ascertain mental health outcomes.”*

Please find the added paragraph on page 29 line 622 :

*“It is worthwhile noting that the measures used to assess outcomes varied across studies. Some used more reliable diagnostic methods, such as ICD codes and DSM-based interviews, while others relied on self-report instruments [36,37,49]. One study used prescription fill data as a proxy indicator, which may be influenced by factors unrelated to policy changes and should therefore be interpreted with caution when evaluating mental health impacts.*

***Comment:***

**-The term ‘hard drugs’ is not preferable in my research area (drug policy) as it can be stigmatising to people who use drugs. There is no clear differentiator between so called ‘soft’ and ‘hard’ drugs, and the term is not preferred by people with lived experience (people who use drugs). I would recommend using the language ‘drugs other than cannabis’, and defining this early on to exclude alcohol and tobacco, etc.

***Response:*** We acknowledge the concerns around the term *"hard drugs"*, and its potential to perpetuate stigma against people who use drugs. In response, we have:

- Removed all references to “hard drugs” from the manuscript and adopted more appropriate and neutral phrasing “non-cannabis psychoactive drugs” “drugs other than cannabis” to describe the category of substances initially targeted in our review.
- Defined this term in the last paragraph of the inroduction to clearly distinguish our focus from studies involving legal substances such as alcohol, tobacco, or prescribed medications.

This is the revised paragraph on page 6 line 202 :

*“The present review expands the scope to include a broader range of drug policies—such as the decriminalization of psychoactive substances beyond cannabis, alcohol, tobacco, or prescribed medications—and extends the analysis period back to January 2001”*

***Comment:***

-93-94 could be revised

***Response:***

We were not entirely sure what specific revision you had in mind for lines 93–94. Once you clarify your suggestion, we will be happy to address it accordingly.

***Comment:***

**-Line 192 – brackets (n = ) should be following the outcome. Eg anxiety (n = 11, depression (n = 21). Appears to be an error somewhere here

***Response:***

The missing word is “psychosis”—the (n=21) refers to the number of studies that focused on psychosis. We have revised the sentence and added the missing outcome.

This is the revised sentence on page 12 line 385 :

*“In terms of mental health outcomes, self-harm including suicide was the most frequently assessed outcome (n = 22), followed by psychosis (n = 21), depression (n = 11), anxiety (n = 9), and psychiatric ED visit/hospitalization (n = 11), as shown in Fig 3.”*

***Comment:***

-B footnote is unclear in table

***Response:***

We have replaced the term “Direction of evidence” with “Overall policy impact” to better reflect the nature of the findings and improve clarity for both qualitative and quantitative studies. We have also revised the corresponding footnotes in the table to clearly define each category—*positive*, *negative*, *mixed*, and *no significant impact*—so that readers can more easily interpret the results.

This is the revised footnote on page 22 line 487 and page 26 line 542 :

*“ ^a^ Overall policy impact on mental health outcomes. A positive impact indicates improvement in mental health symptoms or a reduction in adverse outcomes. A negative impact indicates worsening symptoms or an increase in adverse outcomes. ^b^ No significant impact of the policy on mental health outcomes (i.e., null or inconclusive findings). ^c^ Mixed results were observed across different subgroups or outcome measures.”*

***Comment:***

-Alcohol induced psychosis is included for one paper – but alcohol is noted as excluded

***Response:***

The item has now been removed from the outcome column.
Please find the revised Table 1 on page 14 line 399.

***Comment:***

**-Kim (43) reported as increase, but outcomes state ‘reductions in…’

***Response:***

This issue has been corrected.
Please find the revised Table 1 on page 14 line 399.

***Comment:***

**-Lines 270-272 – needs clarification, error somewhere about Canada vs US

***Response:***

We revised the paragraph to enhance clarity.

Please find the revised table on page 31 line 682:

*“Studies from both the United States and Canada predominantly found that cannabis policies had no significant impact on mental health outcomes, with 34.5% of U.S. studies and 38.9% of Canadian studies reporting no overall effect. However, a greater proportion of U.S. studies reported a negative overall impact, while Canadian studies more frequently indicated mixed findings. A comparison of overall policy impacts between studies from the U.S. and Canada is presented in Figure 6.”*

**Response to Reviewer #2 Comments:**

Overall Feedback:

This paper addresses an important and timely topic, and I commend the authors for their efforts. However, the current version has substantial weaknesses in clarity, definitions, methodology, and framing. The extent of changes required prior to publication suggest that a straightforward revision would not be sufficient. Once you have addressed the comments below, with additional input from co-authors and guidance from subject experts, I would encourage you to re-submit for publication.

Overall, you need to improve your specificity and clarity in communicating; particularly with regards to:

***Comment:***

(1) Define and maintain consistent terminology regarding mental health throughout the paper

***Response:***

We have reviewed the manuscript to ensure that terminology related to mental health is clearly defined and used consistently throughout.
***Comment:***

**(2) It is crucial that you specify what you are including when referring to mental health outcomes, specifically, you need to acknowledge and justify that you are not including substance use disorders and addiction as mental health outcomes. Regardless of whether you are examining this as an outcome of interest, failing to acknowledge them as mental health difficulties risks perpetuating stigma and misunderstanding

***Response:***

We fully acknowledge that substance use disorders (SUDs) are recognized mental health conditions. However, given numerous existing reviews focused specifically on SUDs and their relationship to policy changes and comorbidities, we chose to narrow our focus to non-SUD mental health outcomes. This decision was made to highlight other potential mental health burdens—such as depression, anxiety, and psychosis—that may be associated with substance use but are often overshadowed when SUD is the primary outcome.

In response, we have revised the manuscript to explicitly state that while SUDs are classified as mental health disorders, they were not included as outcomes of interest in this review. This decision was made to focus specifically on substance-related mental health outcomes rather than substance use disorder itself (e.g., depression, anxiety, psychosis) that may arise following drug use or policy changes.

We now clearly justify this scope in the Methods section and acknowledge the exclusion of SUDs as a deliberate analytic boundary rather than an omission of their clinical relevance, to ensures that our framing respects the legitimacy of SUDs as mental health conditions while maintaining the focus of the review.

Please find the revised paragraph on page 9 line 283 :

*“The primary outcome of interest was the onset of non-SUD mental health complications—such as depression or anxiety disorders—that may develop following exposure to psychoactive substances, regardless of whether the individual meets criteria for a substance use disorder. This includes both acute mental health crises triggered by substance use (e.g., psychotic episodes during intoxication) and longer-lasting or recurrent disorders that may persist beyond the immediate effects of the substance [18,33]. We included studies reporting population-level measures derived from national statistics, administrative health records, or large cohort studies. Both large, population-based datasets and smaller clinical or community samples were accepted, provided they met our diagnostic or measurement criteria. Studies were included if they employed clinical diagnoses (based on DSM or ICD criteria), validated diagnostic interviews, prescription-fill records for major psychiatric medications, or standardized self-report scales to ascertain mental health outcomes. We excluded any studies that:*

*•	Reported solely on patterns of drug consumption (e.g., frequency, quantity) or on problematic use indicators (e.g., dependence, abuse) or all-cause drug-related emergency department visits (e.g., overdose rates, drug-related deaths, intoxication, or withdrawal) without measuring psychiatric symptoms;*

*•	Focused exclusively on substance use disorders (SUDs). While SUDs are recognized substance-related mental conditions, they were excluded to allow a focused examination of non-SUD substance-related mental health outcomes—such as depression, anxiety, or psychosis;*

*•	Investigated physical health outcomes, civic and social impacts, motor vehicle accidents, drug-related arrests, law enforcement activity, or any aspect of drug trafficking, manufacturing, or cultivation;*

*•	Addressed mental disorders associated exclusively with prescribed medications, alcohol, tobacco, or caffeine. Prescribed cannabis and psychedelics were treated as distinct exposures, as their medical legalization falls within the scope of this review. Here “prescribed medications” refers specifically to substances commonly used without policy-specific regulation (e.g., benzodiazepines).”*

***Comment:***

**(3) The term "hard drug" is controversial and lacks a universally accepted definition, it is important that you define how you are using the term. Consider specifying which substances are being referred to or using more neutral, precise terms.

***Response:***

We acknowledge the concerns around the term *"hard drugs"*, including its lack of clear definitional boundaries and its potential to perpetuate stigma against people who use drugs. In response, we have:

- Removed all references to “hard drugs” from the manuscript; and adopted more appropriate and neutral phrasing “non-cannabis psychoactive drugs” or “drugs other than cannabis” to describe the category of substances targeted in our review.
- Defined this term in the Inroduction and Methods to clearly distinguish our focus from studies involving legal substances such as alcohol, tobacco, or prescribed medications. And detailed our outcome of interest in the Methods section.

This is the revised paragraph on page 6 line 202 :

*“The present review expands the scope to include a broader range of drug policies—such as the decriminalization, legalization or commercialization of psychoactive substances other than cannabis, alcohol, tobacco or prescribed medications—and extends the analysis period back to January 2001.”*

***Comment:***

**(4) The term “liberal drug policies” is used repeatedly without adequate explanation or recognition of the variation across different contexts.

***Response:***

We have made several revisions to clarify and contextualize this terminology:

- In the Introduction, we have clarified the use of the term “liberal drug policies” by linking it more precisely to the specific policy types examined in this review—namely, decriminalization, legalization, and commercialization.
- We have also added a note in the third paragraph of the Introduction to explicitly acknowledge the role of contextual factors in shaping the outcomes of drug policy reforms across different jurisdictions.
- Additionally, the Methods section now includes a clear outline of the specific policy types covered in our review, along with brief descriptions of each, to better define the scope of our analysis.

Please find the revised paragraph on page 3 line 81 :

*“In recent years, global drug policy has undergone a significant shift, moving away from punitive, prohibitionist approaches toward more reform-oriented models. These “liberal drug policies”—commonly defined as regulatory frameworks that include decriminalization, legalization, and commercialization—aim to reduce criminal penalties, regulate markets, and prioritize public health.”*

Please find the revised paragraph on page 4 line 129 :

*“Drug-*related outcomes—whether beneficial or harmful—are highly dependent on contextual factors such as health system capacity, enforcement practices, public attitudes, and the pre-existin*g status of drug use, treatment infrastructure, and criminal justice involvement within each country or jurisdiction.”*

Please find the revised paragraph on page 8 line 268 :

*“Our initial aim was to assess the effects of liberal policies targeting non-cannabis psychoactive substances (e.g., opioids, stimulants). Here, the “policy framework” encompasses three main regulatory approaches:*

*•	Decriminalization: removal of criminal penalties for possession or personal use, typically replacing them with administrative fines or diversion to treatment*

*•	Legalization: establishment of a lawful pathway for production, distribution, and consumption under government oversight*

*•	Commercialization: introduction of a regulated market in which private entities can produce, market, and sell substances, often alongside taxation and advertising controls*

### *Because our targeted search for studies on substances other than cannabis yielded no eligible papers, we broadened our scope to include evaluations of cannabis policies. Consequently, any study examining the impact of decriminalization, legalization, commercialization, or similar regulatory changes was considered eligible, regardless of substance class.”*

### **Specific Comments** Abstract

### ***Comment:*** Requires significant re-wording and improved specificity.

***Response:***

We have revised the section to improve clarity, specificity, and overall wording to ensure the content effectively communicates the intended message.

Please find the revised abstract on page 2 line 17 :

*“As countries increasingly adopt more liberal drug policies, concerns have emerged about their potential impact on drug-related outcomes, including mental health. Although a strong link exists between problematic drug use and mental health complications—particularly the co-occurrence of mental disorders with substance use disorders (SUDs)—the broader mental health effects of drug policy remain underexplored. This review aims to map existing research on non-SUD mental health outcomes associated with drug decriminalization and legalization policies. We conducted a scoping review following JBI guidelines and the PRISMA-ScR checklist. Studies published between January 2001 and December 2024 were included if they examined non-SUD mental health outcomes related to drug policy changes, particularly decriminalization, legalization, or commercialization. We searched Medline, EMBASE, CINAHL, PsycInfo, and Web of Science, and manually screened relevant policy reports. Only English-language studies were included. Data extraction and analysis were conducted using Covidence, with a descriptive summary of study characteristics and findings. A total of 55 studies met inclusion criteria, comprising 16 review papers and 39 original research articles (37 quantitative and 2 qualitative). Most evidence came from the United States (n = 29) and Canada (n = 18). No studies examined the mental health impacts of non-cannabis psychoactive drug policies or decriminalization frameworks. The most frequently assessed outcomes were psychosis, suicide, and depression. This review maps the current evidence base and identifies major gaps—particularly concerning decriminalization and policies targeting substances other than cannabis. The heterogeneity in study designs and policy contexts highlights the need for multi-faceted, context-sensitive research to inform future drug policy.”*

Introduction

***Comment:***

**Paragraph 1 lacks clarity and requires re-wording to ensure communication of key messages. Define key terms such as "liberal drug policies" to ensure readers understand the framing.

***Response:***

We have revised the first paragraph to improve clarity and ensure that the key messages are communicated more effectively. Specifically, we have:

- Reworded the paragraph to enhance readability and streamline the framing of the review.
- Defined key terms, including “liberal drug policies,” early in the text. Rather than using the term broadly, we now refer specifically to decriminalization, legalization, and commercialization, with clear definitions provided in the Methods section.

Please find the revised paragraph on page 3 line 81 :

*“In recent years, global drug policy has undergone a significant shift, moving away from punitive, prohibitionist approaches toward more reform-oriented models. These “liberal drug policies”—commonly defined as regulatory frameworks that include decriminalization, legalization, and commercialization—aim to reduce criminal penalties, regulate markets, and prioritize public health. To date, over 30 countries have implemented some form of decriminalization to lessen the legal consequences associated with personal drug use [1]. In 2018, the United Nations encouraged countries to adopt more flexible drug policies that align with their own national priorities and contexts [2].”*

***Comment:***

Line 44: Specify UN priorities you are alluding to.

***Response:***

To clarify, the reference was not to specific UN priorities, but rather to the United Nations’ 2018 statement encouraging countries to develop drug policies that align with their own national priorities and contexts. We have revised the sentence to reflect this more accurately and avoid any misinterpretation.

***Comment:***

Line 47: Add references and instead of broadly noting public debate, specify in mainstream narratives this is often people lacking in specialist knowledge including politicians as well as those with differing politically aligned stances. Be specific there is no clear black/white evidence either way and there is poor public understanding which further fuels stigma, misinformation and bias.

***Response:***

We have revised the sentence to more accurately reflect the nature of the public debate and added relevant references. We also highlighted that there is currently no conclusive evidence on the overall public health impacts of liberal drug policies, and that this uncertainty, combined with limited public understanding.

Please find the added paragraph on page 4 line 124 :

*“Mainstream narratives—often influenced by non-expert voices such as politicians, media figures, and ideologically driven commentators— frequently reflect polarized views. The absence of conclusive evidence, combined with limited public awareness, has further fueled misperceptions about the policy impacts [8,9].”*

***Comment:***

Paragraph beginning line 59: Consider acknowledging the contrast with countries that have strict drug policies but high usage rates (e.g., France).

***Response:***

We have revised the paragraph to include a contrast between France and Sweden, which maintain strict drug policies yet show persistent or rising drug-related harms, and Japan, which also enforces strict laws but reports low drug use

Please find the revised text on page 4 line 142 :

*“In contrast, countries such as France and Sweden, which maintain prohibitionist drug policies, have seen persistent or increasing rates of drug use and related harms, suggesting that restrictive laws alone may not effectively curb substance use [14,15]. Japan, on the other hand, also enforces a highly punitive drug policy, but reports low levels of drug use [16]. These contrasts suggests that the effectiveness of drug policy cannot be evaluated in isolation from the broader social, health, and legal context in which it is implemented.”*

***Comment:***

Line 54 and Paragraph beginning line 59: Clarify whether higher use is presented as a negative or neutral outcome. Consider contextualising the increase similarly to other commercially available goods, where greater availability leads to higher consumption?

***Response:***

We have clarified the paragraph to note that increased use following legalization may result from greater availability, which can lead to negative health outcomes.

Please find the revised text on page 3 line 98 :

*“Opponents argue that by increasing the availability of these substances, legalization may lead to higher consumption and, consequently, a rise in negative health outcomes. Advocates, however, contend that legalization enhances control over drug quality, disrupts illegal markets, and generates tax revenue that can be reinvested into public health initiatives [6,7].”*

***Comment:***

**Paragraph beginning line 71 (and overall) - The omission of addiction and substance use disorders is significant and needs addressing. Additionally, there is no acknowledgement that mental health issues are associated with substance use - the bi-directional relationship - or the global trends in worsening mental health.

***Response:***

We have revised the intoduction to highlight the bidirectional relationship between substance use and mental health, distinction between SUD and recreational drug use, and the broader context of global mental health trends.

Please find the revised paragraphs on page 5 line 154 :

*“Beyond legal and economic effects, liberal drug policies may have profound implications for public mental health. Substance use and mental health share a complex, bidirectional relationship: each can precipitate or aggravate the other [17]. Although the primary mental health consequence of problematic drug use is the development of substance use disorders (SUDs), drug exposure can also induce a spectrum of other psychiatric symptoms, including depression, anxiety, and psychosis [18]. Even occasional or nondependent consumption may provoke mental health issues [19], yet these co-occurring complications are particularly prevalent among individuals with SUDs [20].*

*The nature and severity of these mental health effects depend on the type of substance, the pattern of use, and individual vulnerability. For example, early and heavy cannabis use in adolescence can disrupt neural maturation and heighten the risk of psychosis, depression, and suicidal ideation [21]. Problematic use of opioids—whether prescribed or illicit—are linked to depression, anxiety, and suicidality, especially during withdrawal phases [22,23]. Stimulants such as cocaine and amphetamines may trigger acute paranoia and psychotic episodes, with chronic use often resulting in persistent cognitive deficits [24,25]. Harmful use of legally available substances like alcohol use disorder can also elevate the risks of depressive and suicide [26]. These overlapping conditions not only complicate diagnosis and treatment but also point to shared biological and psychosocial vulnerabilities.*

*Despite clear associations, the direction of causality remains under debate. It remains unresolved whether escalating substance use worldwide is driving the surge in mental health disorders, or whether worsening psychological well-being is pushing more individuals toward self-medication. In this context, evaluating how drug policy—be it legalization, decriminalization, or harm-reduction strategies—modulates these intertwined outcomes is an essential focus for future research.”*

***Comment:***

**Line 85 – Ultimately the review covers the same focus as Fortier et al, and it includes the same studies, the rationale needs strengthening for what this review adds – does the paper offer any new insights?

***Response:***

We have clarified our rationale for how our review adds to the work by Fortier et al., which included only 28 original studies. We highlighted that our review integrates findings from both original studies and existing reviews, allowing us to provide a more comprehensive synthesis of the available evidence. Also, we explained that our review broadens the policy scope beyond cannabis-related policies to cover regulations targetting other psychoactive substances. Additionally, we extended the timeframe back to January 2001 to capture both early policy changes and more recent developments.

Please find the revised paragraph on page 6 line 197 :

*“While the issue is pressing, studies exploring the mental health impact of changing drug policies remain sparse. A recent scoping review by Fortier et al. investigated the mental‑health impacts of cannabis legalization [27], identifying twenty‑eight original studies and reports from January 1, 2012, to April 30, 2023. This paper has outlined the limited research available and highlighted the largely inconclusive findings, however, its focus was specifically on cannabis policies.*

*The present review expands the scope to include a broader range of drug policies—such as the decriminalization, legalization or commercialization of psychoactive substances other than cannabis, alcohol, tobacco, or prescribed medications—and extends the analysis period back to January 2001. We also incorporate review papers alongside original studies to map the entire body of existing literature. This approach offers a more comprehensive, up‑to‑date overview of current knowledge.”*

Methods

***Comment:***
Consider whether the absence of consultation with experts by experience, advocates, or third-sector specialists (e.g., Canadian Mental Health Association) is a limitation worth addressing. This could enrich the paper’s perspective and also provide some much-needed guidance on language.

***Response:***

We agree that including perspectives from experts and organizations would have improved the review and helped inform more inclusive and sensitive language. However, this project was conducted without funding, which limited our capacity to engage in formal consultations. We acknowledge this as a limitation in the revised manuscript and recognize the importance of incorporating such voices in future research to enhance relevance, equity, and impact.

Please find the added text on page 35 line 851 :

*“Additionally, our review did not involve consultation with individuals with lived experience, third-sector or governmental organizations, or experts by experience. Engaging these groups in future research could help include diverse perspectives and improve the scrutiny of drug policy impacts in relation to specific settings, conditions, and populations.”*

***Comment:***

Publication type: The exclusion of non-English studies is a notable limitation, particularly given that many countries implementing decriminalization policies are non-English speaking. Acknowledge the study is by default excluding all localised articles and policy reports.

***Response:***

This point is acknowledged in our limitations section, where we note the potential underestimation of evidence from regions such as Europe and South America. We added a note to recognize that this language restriction excludes localized articles and policy reports that could offer valuable contextual insights.

Please find the revised text on page 35 line 846 :

*“We acknowledge that the exclusion of non-English literature may have led to an underestimation of evidence from certain countries, particularly in Europe and South America, and excludes localized articles and policy reports published in non-English media that may offer important context in regions where decriminalization policies have been implemented.”*

***Comment:***

**Intervention: As noted previously, define what is meant by "hard drugs". Additionally, how are you differentiating prescribed medications (line 146) from prescribed cannabis (which is a medicine)? or prescribed psychedelics (in the case of the legalisation of psychedelic therapies)?

***Response:***

We have clarified our categorization of substances in the revised manuscript:

We have replaced the term “hard drugs” with more precise language, using “non-cannabis psychoactive drugs” or “drugs other than cannabis” to more accurately reflect the scope of substances addressed in our review, including opioids, cocaine, and amphetamines, while avoiding stigmatizing terminology.

Additionally, we recognize the need to clearly differentiate among prescribed substances. Since the mental health impacts of medical legalization are within our scope of interest, we have chosen to exclude medical cannabis and psychedelics from the general “prescribed medications” category and included them in our review. The “prescribed medications” category in our review continues to include substances such as benzodiazepines and other psychoactive drugs commonly prescribed in clinical practice but not tied to specific legalization or policy reform efforts. These clarifications are now reflected in the Methods section.

Please find the added text on page 10 line 314 :

*“We excluded studies that: ..addressed mental disorders associated exclusively with prescribed medications, alcohol, tobacco, or caffeine. Prescribed cannabis and psychedelics were treated as distinct exposures, as their medical legalization falls within the scope of this review. “Prescribed medications” refers specifically to substances commonly used without policy-specific regulation (e.g., benzodiazepines).”*

***Comment:***

**Outcome: Clarify the definition of secondary mental health disorders. Are these considered secondary to substance use (which is not the same as substance use disorder and addiction), or mental health crises triggered by substance use? Are you differentiating between one-time and chronic effects?

***Response:***

We have removed the term “secondary mental health disorders” and clarified our definition of our outcome of interest in the revised methods section, and included both transient and chronic presentations where studies provide that level of detail.

Please find the revised text on page 9 line 283 :

*“The primary outcome of interest was the onset of non-SUD mental health complications—such as depression or anxiety disorders—that may develop following exposure to psychoactive substances, regardless of whether the individual meets criteria for a substance use disorder. This includes both acute mental health crises triggered by substance use (e.g., psychotic episodes during intoxication) and longer-lasting or recurrent disorders that may persist beyond the immediate effects of the substance [18,33].”*

***Comment:***

Exclusion: Expand and justify the exclusion of studies examining substance use disorder outcomes (given that this is a mental health condition).

***Response:***

We fully acknowledge that substance use disorders (SUDs) are recognized mental health conditions. However, given numerous existing reviews focused specifically on SUDs and their relationship to policy changes and comorbidities, we chose to narrow our focus to non-SUD mental health outcomes. This decision was made to highlight other potential mental health burdens—such as depression, anxiety, and psychosis—that may be associated with substance use but are often overshadowed when SUD is the primary outcome.

In response, we have revised the manuscript to explicitly state that while SUDs are classified as mental health disorders, they were not included as outcomes of interest in this review. This decision was made to focus specifically on substance-related mental health outcomes rather than substance use disorder itself (e.g., depression, anxiety, psychosis) that may arise following drug use or policy changes.

We now justify this scope in the Methods section and acknowledge the exclusion of SUDs as a deliberate analytic boundary rather than an omission of their clinical relevance, to ensures that our framing respects the legitimacy of SUDs as mental health conditions while maintaining the focus of the review.

This is the revised paragraph on page 10 line 307 :

*“We excluded studies that: ...focused exclusively on substance use disorders (SUDs). While SUDs are recognized substance-related mental conditions, they were excluded to allow a focused examination of non-SUD substance-related mental health outcomes—such as depression, anxiety, or psychosis;”*

Results

***Comment:***

Line 191: Distinguish between self-harm and suicide – specify, do you mean suicidal ideation, survival or deaths by suicide? Categorise appropriately.

***Response:***

In many of the included studies, specific details distinguishing between suicidal ideation, non-fatal self-harm, and deaths by suicide were not consistently reported. For this reason, we chose to group these outcomes under the broader category of “self-harm or suicide” in the main text to ensure consistency across studies. However, where more specific information was available, we have provided those details in the results table for greater clarity.
**Line 192: What is the (n=21) referring to? Re-word for clarity.

***Response:***

The missing word is “psychosis”—the (n=21) refers to the number of studies that focused on psychosis. We have revised the sentence and added the missing outcome.

This is the revised sentence on page 12 line 385 :

*“In terms of mental health outcomes, self-harm including suicide was the most frequently assessed outcome (n = 22), followed by psychosis (n = 21), depression (n = 11), anxiety (n = 9), and psychiatric ED visit/hospitalization (n = 11), as shown in Fig 3.”*

***Comment:***

**Table 1: Define policy framework categories earlier in the paper for clarity.

***Response:***

We have now added a clear definition of our “policy framework,” described how we defined categories.

Please find the revised paragraph on page 8 line 268 :

*“Our initial aim was to assess the effects of liberal policies targeting non-cannabis psychoactive substances (e.g., opioids, stimulants). Here, the “policy framework” encompasses three main regulatory approaches:*

*•	Decriminalization: removal of criminal penalties for possession or personal use, typically replacing them with administrative fines or diversion to treatment*

*•	Legalization: establishment of a lawful pathway for production, distribution, and consumption under government oversight*

*•	Commercialization: introduction of a regulated market in which private entities can produce, market, and sell substances, often alongside taxation and advertising controls ”*

***Comment:***

Time trends: Given that you only include English papers, it follows, there are no papers from non-English speaking countries, so reference to Portugal here does not make sense; it would be more relevant to refer to the dates of decriminalisation in the locations of the studies included in the analysis.

***Response:***

We have revised the text to remove the reference to Portugal and have instead included relevant dates of drug policy developments in the United States and Canada.

Please find the revised paragraph on page 27 line 561 :

*“Our search covered a 24-year period from January 2001 through the end of 2024. However, as shown in Figure 4, all included studies were published within the past 10 years, with none identified prior to 2014. This highlights a notable gap in the literature, especially considering that cannabis decriminalization in the United States began as early as the 1970s, followed by the legalization of medical cannabis in California in 1996 [89] and the introduction of medical cannabis legislation in Canada in 2001[90].”*

**Summary of original studies:

***Comment:***

Address the disparity in outcome indicators — for example, psychiatric medication fill rates may reflect prescribing trends rather than psychiatric symptomatology, making them less comparable to direct measures of mental health outcomes like depression.

***Response:***

We have addressed the variation in outcome measurement tools and highlighted the lower comparability and reliability of indirect assessment methods, such as prescription fill data.

Please find the added text on page 29 line 622 :

*“It is worthwhile noting that the measures used to assess outcomes varied across studies. Some used more reliable diagnostic methods, such as ICD codes and DSM-based interviews, while others relied on self-report instruments [36,37,49]. One study used prescription fill data as a proxy indicator, which may be influenced by factors unrelated to policy changes and should therefore be interpreted with caution when evaluating mental health impacts.”*

***Comment:***

Clarify comparison/control groups (e.g., administrative data before/after policy implementation) and add mention of trends based on the type of study.

***Response:***

We have added a column to Table 1 to specify the control or comparison groups used in each study (e.g., pre-policy periods, regional comparisons). We also included a paragraph summarizing key observations related to these comparison groups.

Please find the added text on page 28 line 606 :

*“The majority of studies (n=24) compared pre- and post-legalization trends, primarily using quasi-experimental (e.g., interrupted time series) and longitudinal designs. Fifteen studies compared states or regions that adopted the policy with those that did not, either alongside a pre-post approach or as a standalone comparison. Two studies constructed synthetic control groups to represent counterfactual scenarios in which the policy had not been implemented [39,45]. Additionally, two studies used conditions or emergency department visits unrelated to cannabis as comparison outcomes [42,51].”*

***Comment:***

Comment on the inclusion of the UK despite its lack of liberal drug policies.

***Response:***

The inclusion of the study from the UK was based on its relevance to our inclusion criteria, as it examined mental health outcomes in the context of a limited 2018 policy change allowing the prescription of cannabis for medical purposes. We have added a note of this in the manuscript.

Please find the added text on page 27 line 578 :

*“We identified one study from the United Kingdom, where a liberal drug policy is not broadly implemented and only allows restricted cannabis prescriptions for medical purposes[40].”*

***Comment:***

Consider also summarising trends relative to the policy frameworks examined.

***Response:***

This has been addressed in the manuscript under the section “Time Trends and Policy Frameworks,” where we highlight trends in types of policy frameworks studied in the included papers.

Please find the paragraph on page 27 line 567 :

*“Among the 55 reviewed papers, 29 evaluated the effects of recreational legalization, with or without including the impact of opening commercial dispensaries; 7 focused only on medical legalization; and 18 analyzed the impacts of both medical and recreational laws. Thirteen studies considered commercialization and opening of the cannabis dispensaries in their analyses. One review paper only examined the literature on commercialization and access to cannabis retailers[76]. Notably, none of the original studies or review papers directly examined decriminalization as a distinct drug policy framework.”*

***Comment:***

Summary of review papers: Include a narrative synthesis of the findings, similar to the original studies section.

***Response:***

We have expanded this section to provide additional detail and depth to the narrative synthesis.

Please find the paragraph on page 29 line 628 :

*“Table 2 presents a summary of the included review papers. Sixteen review papers were included, comprising nine narrative reviews [64,66,67,69–72,74,76], six systematic reviews[14,63,65,68,73,75] and one scoping review[20]. Half of the reviews looked over papers published globally[20,63,65,67,68,73,75], one third specifically focused on the United States[66,69,70,72,76], and the remaining reviewed the policy impact in Canada[14,64,71,74]. Notably, 20 of the original studies included in our scoping review were also examined in one or more of the included review papers. This overlap reflects the limited pool of available evidence in this area and has been specified in Table 2 to acknowledge repetition and ensure transparency in the interpretation of findings.*

*Most of the review papers examined a broader range of outcomes beyond mental health complications. Drug use patterns, overdose rates, SUD prevalence, cannabis-related traffic incidents and physical health issues were among the outcomes analyzed, alongside non-SUD mental health outcomes. Among these, psychosis was the most commonly explored non-SUD mental health outcome [14,20,65,68,71–75], followed by, suicide or self-harm[20,65,69–72,75,76], psychiatric ED visit or hospitalization[14,63,64,66–68,70,74], depression[20,71,72], anxiety[20,68,71], and SMI[69,77] (See Fig 3).”*

***Comment:***

Clarify which original studies have also been included in these reviews to acknowledge repetition through secondary interpretation.

***Response:***

We have added a column to the review papers table to specify which original studies were included in each review. Additionally, we have noted in the narrative description that 20 of the original studies included in our scoping review were also reviewed in one or more of the included review papers, to acknowledge repetition.

Please find the revised table on page 22 line 490.

Please find the added text on page 29 line 633:

*“Notably, 20 of the original studies included in our scoping review were also examined in one or more of the included review papers. This overlap reflects the limited pool of available evidence in this area and has been specified in Table 2 to acknowledge repetition and ensure transparency in the interpretation of findings.”*

***Comment:***

**Direction of evidence (Lines 270 and 272): The phrasing is inconsistent — clarify whether Canadian studies predominantly support or contrast with US findings.

***Response:***

We revised the paragraph to enhance clarity.

Please find the revised table on page 31 line 682 :

“Studies from both the United States and Canada predominantly found that cannabis policies had no significant impact on mental health outcomes, with 34.5% of U.S. studies and 38.9% of Canadian studies reporting no overall effect. However, a greater proportion of U.S. studies reported a negative overall impact, while Canadian studies more frequently indicated mixed findings. A comparison of overall policy impacts between studies from the U.S. and Canada is presented in Figure 6.”

Discussion

***Comment:***

Line 281: Ensure consistent terminology (e.g., "concurrent", “secondary”). As noted previously ensure consistent language regarding mental health throughout.

***Response:***

We have removed the terms “secondary” and “concurrent” and now consistently refer to the outcomes as “non-SUD mental health complications” or “co-occurring mental disorders,” as appropriate. The difinitions have been provided in the methods section.

Please find the text on page 9 line 283 :

*“The primary outcome of interest was the onset of non-SUD mental health complications—such as depression or anxiety disorders—that may develop following exposure to psychoactive substances, regardless of whether the individual meets criteria for a substance use disorder. This includes both acute mental health crises triggered by substance use (e.g., psychotic episodes during intoxication) and longer-lasting or recurrent disorders that may persist beyond the immediate effects of the substance (Baldacchino 2021, Torrens 2010).”*

Please find the revised text on page 31 line 690 :

*“This scoping review aimed to provide a landscape of the existing evidence on the impact of liberal drug legislation frameworks on non-SUD mental health outcomes and the prevalence of co-occurring mental conditions.”*

***Comment:***

Line 283: Expand on what is meant by "sometimes unreliable," providing concrete examples.

***Response:***

We agree that "unreliable" was not the most appropriate term and have removed it from the text. Instead, we have revised the sentence to emphasize that findings are often shaped by local context, limiting their generalizability across different jurisdictions.

Please find the revised text on page 31 line 692 :

“The existing literature on the mental health impacts of liberal drug policies is limited and often inconsistent, with findings often shaped by local context, making them difficult to generalize across jurisdictions.”

***Comment:***

Line 303: Clarify that while there may be a public misperception of cannabis use being a ‘gateway’ to using more harmful substances, there is no conclusive evidence supporting this causality.

***Response:***

We have clarified the text to note this.

Please find the revised text on page 32 line 733 :

*“Cannabis is often cited by this group as a “gateway drug,” based on the perception that its relative safety could lead individuals to experiment with more harmful substances such as opioids and stimulants [6,80]. However, it is important to note that there is no conclusive evidence supporting a causal relationship between cannabis use and the use of more dangerous substances (Noel 2018).”*

***Comment:***

Consider commenting on the variability in supply safety and differing policy frameworks e.g. the risks associated with high-potency THC.

***Response:***

We have added a note in the discussion to acknowledge that variability in supply safety can influence mental health outcomes.

Please find the revised text on page 35 line 838 :

*“These examples underscore that drug policies are not uniform interventions: local enforcement practices, health-system capacity, and complementary regulations can strongly influence psychiatric outcomes. It is also important to consider that these outcomes may be influenced by variability in supply safety and regulatory frameworks—such as differences in product potency, quality control, and access. For instance, the availability of high-potency THC products in some jurisdictions may elevate the risk of psychosis and other adverse psychiatric effects [71], underscoring the need to interpret findings within the context of specific policy and regulatory environments.”*

***Comment:***

Lines 363: Acknowledge that this review is not global, that it is only inclusive of English-speaking countries, focusing primarily on North American policies.

***Response:***

This point has been acknowledged in the limitations section of the manuscript, where we note that the review is not global due to the exclusion of non-English-language studies.

Please find the paragraph on page 35 line 846 :

*“We acknowledge that the exclusion of non-English literature may have led to an underestimation of evidence from certain countries, particularly in Europe and South America, and excludes localized articles and policy reports published in non-English media that may offer important context in regions where decriminalization policies have been implemented.”*

***Comment:***

Line 366: Offer specific recommendations for improving study designs, addressing gaps and expanding research - avoid vague or repetitive conclusions.

***Response:***

We have revised the discussion section to include more specific and actionable recommendations for future research.

Please find the revised text on page 36 line 864 :

*“To generate evidence that is both generalizable and policy-relevant, future studies must move beyond basic outcome assessments and adopt designs that account for broader policy and social contexts. This includes pre-policy conditions, the scale of harm reduction and public health efforts, implementation funding, and the availability of mental health and addiction services. Furthermore, studies should incorporate longer follow-up periods to capture both the immediate and long-term effects of policy changes, as some mental health outcomes may emerge or evolve well after the initial policy implementation.*

*Importantly, future research should also address the clear gap in evaluations of policy reforms targeting substances other than cannabis. Qualitative and mixed-methods approaches—incorporating the lived experiences of people who use drugs, community members, service providers, and policymakers—can reveal unintended consequences, community dynamics, and contextual factors shaping policy impact. By integrating these perspectives, research can better explain cross-jurisdictional variation and support the development of more nuanced, equitable, and effective drug policies.”*

***Comment:***

Line 369: Remove the phrase "especially in regions with a long history of such policies," this is inappropriate given that the review design inevitably excludes these countries by excluding non-English content.

***Response:***

We agree with your observation and have removed the phrase.

Please find the revised text on page 36 line 861 :

*“These findings underscore the critical need for more comprehensive and methodologically rigorous research to deepen our understanding of the mental health impacts of less punitive drug policies.”*

***Comment:***

Additional query: Generation of the plots is a substantial contribution, unless personal preference, Hamidreza Aftabi should be a named author.

***Response:***

Thank you for this thoughtful suggestion. The plots were generated by the lead author, with Mr. Hamidreza Aftabi providing assistance on coding and troubleshooting when needed. As his background is in computer engineering and he has not been involved in the conceptual, analytical, or interpretive aspects of the review, he has chosen not to be listed as a co-author and has expressed no interest in authorship.
