# Supplementary material for: Exploring the impact of drug decriminalization and legalization policies on mental health outcomes: A scoping review
Source: PLOS Ment Health. 2025 Oct 15;2(10):e0000358. doi: 10.1371/journal.pmen.0000358 (PMC12798171; doi:10.1371/journal.pmen.0000358)
Supplement: Attachment — Submitted filename: Rebuttal_letterPLOS Mental Health_Sep_14.docx [file pmen.0000358.s006.docx]

**September 14, 2025**

Dear Prof. Cumming,
Academic Editor, PLOS Mental Health

We are grateful to you and the reviewers for the additional feedback provided on our revised manuscript. We have carefully addressed these points, and the manuscript now uses more consistent terminology and used more precise wording to avoid overstating or understating key notions.

Enclosed with this letter is our revised manuscript (PMEN-D-25-00024R1), entitled “Exploring the Impact of Drug Decriminalization and Legalization Policies on Mental Health Outcomes: A Scoping Review.” A detailed, point-by-point response to each comment is also attached.

We once again thank the editorial team and reviewers for their constructive input and hope that the revisions now satisfactorily address all concerns and align with the standards of PLOS Mental Health.

Sincerely,

On behalf of the authors,

Mana Mohebbian,

PhD Candidate,

University of British Columbia

**Point-by-point responses**

Reviewer #2: Thank you for your integration of reviewer comments, it has significantly improved the paper. However, there remains a number of clarifications required prior to publication. Several sections use language that seems to imply causality, understate key advocacy arguments, and inconsistently frame mental health in relation to substance use. Addressing these issues will improve clarity, provide a more balanced and nuanced framing, aligning with existing evidence.

Throughout:

**Comment:**

The mental health language is not consistent throughout - you switch between ‘mental health complications’ ‘mental health disorders’ ‘mental health’ as well as outdated terms ‘mental disorders’ ‘mental conditions’ etc. Health Canada often uses "mental health conditions". Pick one term and use it consistently throughout.

**Response:**

We have revised the manuscript to replace alternative terms with “mental health conditions” for consistency.

**Comment:**

Throughout the paper, I believe some of the language is misleading, inferring causality without evidence nor reflective of the included studies. For example, the phrasing “substance-related mental health outcomes” implies a causal relationship however this is problematic given the bi-directional nature of the relationship and that the included studies are not causal analyses. Consider "mental health conditions in the context of substance use".

**Response:**

To avoid implying causality, we have revised the wording where possible to use the suggested term “mental health conditions in the context of substance use.”

Abstract:

**Comment:**

The current framing implies mental health is one subset of "drug-related outcomes, when mental health is central to understanding both the causes and consequences of drug use. Consider acknowledging the complex relationship upfront e.g. "concerns have emerged about their broader health and social impacts, recognizing the complex bidirectional relationship between mental health and substance use".

**Response:**

We have revised the abstract to explicitly acknowledge the complex and reciprocal relationship between mental health and substance use, thereby strengthening the framing and clarity of our rationale.

Please see the revised abstract on page 2 line 18.

*“As countries increasingly adopt more liberal drug policies, concerns have emerged about their broader health and social impacts. A complex bidirectional relationship exists between problematic drug use and mental health conditions. This is particularly evident in the co-occurrence of mental health disorders with substance use disorders (SUDs). However, the broader mental health effects of drug policy remain underexplored.”*

Introduction

**Comment:**

Line 44, re: UN priorities – I believe "encouraging more flexible drug policies" misrepresents the UN position document, perhaps quote the UN document directly.

**Response:**

We have revised the sentence to reflect the language used in the original UN document. The sentence now quotes directly from the relevant UNODC policy paper to ensure alignment with the source and to avoid mischaracterizing the organization's stance.

Please see the revised text on page 3 line 54.

*“In 2018, the United Nations guidance has emphasized the importance of aligning drug policy with public health, development, and human rights objectives, noting that countries should ‘place people, health and human rights at the center’ of drug policy responses [2].”*

**Comment:**

Line 56-63, the summary of advocates’ arguments is incomplete, centring on economic and regulatory control arguments, however, advocates typically place greater emphasis on mortality reduction and harm minimisation. Advocates primarily argue that legalisation/decriminalisation has a public health benefit and saves lives - central advocacy arguments include: preventing overdose deaths through supervised consumption and a regulated supply, expanding harm-reduction services, shifting from criminalisation to treatment and avoiding incarceration-related trauma. Economic benefits are generally framed as secondary to the public health objective of saving lives. Accurately reflecting these priorities is important for a balanced portrayal of both perspectives.

**Response:**

We have revised the relevant section to emphasize on advocacy priorities, including mortality reduction, harm minimisation, and human rights. The updated text now balances both the public health rationale and the economic/regulatory arguments for reform.

Please see the revised text on page 3 line 68:

*“Advocates, however, primarily emphasize the public health benefits of legalization and decriminalization, arguing that these policies can save lives by preventing overdose deaths, and through supervised consumption and regulated supply, expanding harm-reduction and treatment services, and avoiding incarceration-related trauma by shifting from criminalization to care(Barry 2019).”*

**Comment:**

Line 83-90, this paragraph continues to frame mental health as primarily a consequence of drug use rather than acknowledging that mental health and trauma are often primary drivers of substance use. Strengthening discussion of mental health, trauma, adverse childhood experiences as drivers would create a more balanced and evidence-based framing.

**Response:**

The paragraph has been revised to better reflect upstream factors, such as childhood experiences and poor mental health, as important contributors to substance use initiation and escalation.

Please see the revised text on page 5 line 107:

*“Substance use and mental health share a complex, bidirectional relationship: each can precipitate or aggravate the other [17]. Importantly, poor mental health, trauma, and adverse childhood experiences (ACEs) are recognized as significant upstream drivers of substance use initiation and escalation(Stewart, 2023). Individuals exposed to ACEs, untreated psychological conditions, or chronic stress are at substantially higher risk of developing substance use disorders (SUDs) and related harms(Grummitt, 2022). At the same time, problematic drug use can contribute to a range of psychiatric symptoms, including depression, anxiety, and psychosis [18].”*

**Comment:**

Line 97, correct wording “Harmful use of legally available substances like alcohol *use disorder* can also elevate the risks of depressive and suicide”

**Response:**

We have revised the sentence and corrected the errors.

Please see the revised text on page 6 line 132:

*“Harmful use of legally available substances like alcohol can also elevate the risks of depression and suicide [26].”*

**Comment:**

Line 101, clarify wording “Despite clear associations, the direction of causality remains under debate.” - the causal relationship between mental health/trauma/Adverse Childhood Experiences on increased risk of substance use and dependency is well-established in the literature.

**Response:**

To improve clarity, we revised the sentence to specify the relationships under discussion.

Please see the revised text on page 6 line 135:

*“Despite strong associations, it remains uncertain whether escalating substance use worldwide is contributing to drug-related mental health complications or whether worsening psychological well-being is driving more individuals to self-medicate with substances.”*

Methods:

**Comment:**

Line 187, “While SUDs are recognized substance-related mental conditions” correct wording “are recognised mental *health* conditions”

**Response:**

We have revised the wording.

Please see the revised text on page 10 line 241:

“*While SUDs are recognized substance-related mental health conditions.*”

**Comment:**

Line 170-198, the outcome definition requires further clarification. The phrase "may develop following exposure" implies causation, but the study designs included cannot establish this, which largely show associations or temporal correlations or qualitative perspectives. Consider something like "non-SUD mental health conditions among populations with substance exposure, recognising that these conditions may be pre-existing, co-occurring, precipitated, or influenced by substance use." This will ensure the outcome definition reflects the methodological limitations of included studies and avoids overstating causal relationships.

**Response:**

We have revised the outcome definition to avoid implying causality and to better reflect the methodological limitations of the included studies.

Please see the revised text on page 9 line 218:

*“The primary outcome of interest was non-SUD mental health conditions such as depression or anxiety disorders among populations with exposure to psychoactive substances, regardless of whether the individual meets criteria for a substance use disorder. These conditions may be pre-existing, co-occurring, precipitated, or influenced by substance use.”*

Results:

**Comment:**

Line 241, remove “Surprisingly”, this would be more appropriate in the discussion.

**Response:**

We have removed the word *“Surprisingly”* from the results section to maintain a neutral and objective tone

Please see the revised text on page 12 line 298:

*“We could not locate any studies examining the impact of policies decriminalizing or legalizing ‘non-cannabis psychoactive drugs’, including substances other than cannabis, alcohol and tobacco, on mental health outcomes (n = 0).”*

**Comment:**

Line 306, grouping suicidal ideation, non-fatal self-harm, and suicide deaths under 'self-harm or suicide' conflates distinct phenomena with different prevalence rates, risk factors, and policy implications - please consider providing a breakdown where data permits and please label the inclusion of suicidal ideation as this is often reported without planning, intent or action, with much wider prevalence than self-harm, survival or deaths by suicide. Conflating these outcomes risks obscuring important differences in prevalence, etiology, and prevention strategies

**Response:**

We have revised the result section to present papers on suicidal ideation, intentional self-harm, and suicide deaths separately, to avoid conflating outcomes.

Please see the revised text on page 28 line 368:

*“Suicide and self-harm (including suicide deaths [38,42,47,48,53,55,59,60,70,71], intentional self-harm [46,48,61,67], and suicidal ideation [69]), along with psychosis[41,45,50,57,58,64–66,72,73,75,76] and depression[40,41,51,52,56,63,69,75] were the most frequently evaluated non-SUD mental health outcomes.”*

**Comment:**

Line 360, “However, a greater proportion of U.S. studies reported a negative overall impact, while Canadian studies more frequently indicated mixed findings.” - remove ‘however’ and please include proportions in text for clarity.

**Response:**

We have removed *“However”* and revised the sentence to include the specific proportions of U.S. and Canadian studies and enhance the clarity.

Please see the revised text on page 31 line 428:

*“The second most common finding in U.S. studies was a negative overall impact (31%), whereas in Canadian studies the second most frequent was mixed finding (33.3%).”*

Discussion:

**Comment:**

Line 383-388, The wording “extreme supporters” reads as bias in framing (the same language is not used for opponents) – please ensure language is consistent and balanced.

**Response:**

We have removed the word *“extreme”* to ensure balanced language when describing both supporters and opponents.

Please see the revised text on page 32 line 456:

*“Supporters of liberal drug regulations view these changes as a promising avenue to reduce criminal charges against drug users and redirect them to health and treatment services. This approach, they argue, will alleviate the social, mental, and physical burdens on individuals, the government, and the justice system [93].”*

**Comment:**

Line 387-388, As above "social, mental, and physical burdens" misses the primary argument about saving lives/reducing mortality.

**Response:**

We have revised the wording to highlight the primary argument regarding saving lives and reducing mortality, while still acknowledging social, mental, and physical burdens.

Please see the revised text on page 32 line 458:

*“This approach, they argue, will reduce overdose deaths and, in turn, alleviate associated social, mental, and physical burdens on individuals, the government, and the justice system [93].”*

**Comment:**

Line 394, “Our review of the papers revealed that differing viewpoints are also reflected in existing research.” - this is not described in your results, is this your subjective impression or is this the framing used by the authors of the papers? Please provide this breakdown in the results section.

**Response:**

We have revised the statement to clarify that this reflects our interpretation of the literature, rather than an unreported result or an explicit framing by the authors.

Please see the revised text on page 33 line 468:

*“We interpret the reviewed papers reflecting differing viewpoints within existing research. These potential biases may have contributed to the mixed and sometimes contradictory findings, making it challenging to draw definitive conclusions.”*

**Comment:**

Line 434-442, captures the complexity required and is a good example of the nuanced analysis the paper should apply throughout.

**Response:**

We are pleased that the nuanced analysis in this section was well received.

**Comment:**

Line 447, this wording is potentially misleading - as it doesn't acknowledge that regulation provides potency testing, labeling, and consumer choice that allows for informed decision-making. While unregulated markets have unpredictable and untested potency levels, regulated markets enable consumers to select products appropriate to their tolerance and preferences, potentially reducing adverse effects through better information and product diversity. For example see research in UK on very high THC levels with very low CBD in street cannabis or the fentanyl crisis also exemplifies how unregulated markets trend toward extremely high-potency substances with no quality control or consumer information, leading to unpredictable and often fatal outcomes.

**Response:**

We have revised the paragraph to address the reviewer’s points by incorporating the role of potency testing and labeling. We also incorporated examples from the UK on high-THC cannabis and one from British Columbia on fentanyl contamination in illicit supplies.

Please see the revised text on page 35 line 525:

*“Weak or absent regulations often result in untested, high-potency substances lacking quality standards or consumer information. For example, in the UK, street cannabis often contains high THC with little CBD, a profile linked to heightened risk of psychosis[108]. Likewise, in British Columbia, illicit opioids are dominated by fentanyl and benzodiazepines, creating a volatile and hazardous supply[109]. Together, these cases show how unregulated markets produce unpredictable, high-potency drugs with serious public health consequences.”*

**Comment:**

Line 468-474, the recommendations are much improved consider including recommendations for study designs that take into account temporality - the current designs can show associations and correlations but can't rule out that observed mental health outcomes preceded substance use or policy changes as such cannot speak to causality.

**Response:**

We have expanded the recommendations to emphasize the importance of study designs that account for temporality. Specifically, we now note that future research should incorporate longer follow-up periods to clarify whether mental health outcomes precede substance use or policy changes, thereby strengthening causal inferences beyond simple associations.

Please see the revised text on page 36 line 560:

*“Importantly, study designs should address temporality by incorporating longer follow-up periods. Current approaches often demonstrate associations without clarifying whether mental health outcomes preceded substance use or policy changes, and some effects may only emerge or evolve well after policy implementation.”*
