# Supplementary material for: Exploring the impact of drug decriminalization and legalization policies on mental health outcomes: A scoping review
Source: PLOS Ment Health. 2025 Oct 15;2(10):e0000358. doi: 10.1371/journal.pmen.0000358 (PMC12798171; doi:10.1371/journal.pmen.0000358)
Supplement: Attachment — Submitted filename: Rebuttal letter_PLOS Mental Health_Sep_22.docx [file pmen.0000358.s007.docx]

**September 22, 2025**

Dear Prof. Cumming,
Academic Editor, PLOS Mental Health

We sincerely appreciate your insightful comments on our revised manuscript. We have carefully addressed each point, clarifying the discussions and recommendations, and moderating the tone to ensure greater precision and professionalism.

Enclosed with this letter is our revised manuscript (PMEN-D-25-00024R2), entitled *“Exploring the Impact of Drug Decriminalization and Legalization Policies on Mental Health Outcomes: A Scoping Review.”* A detailed, point-by-point response to the comments is also attached.

We are grateful to you and the editorial team for your thoughtful feedback and the time dedicated to reviewing our work. We hope that our revisions satisfactorily address all concerns and align with the standards of PLOS Mental Health.

Thank you again for your time and consideration.

Sincerely,

On behalf of the authors,

**Mana Mohebbian,**

PhD Candidate,

University of British Columbia

**Point-by-point responses:**

**Comment:**

p.35 line 525, benzodiazepines are distinct from opioids. Are you meaning to say that benzodiazepines are being misleadingly sold as opioids, or perhaps are being mixed with opioids that are being sold? Also, you mention “a volatile and hazardous supply”. This language is quite emotive, I suggest more plain language that explains that high potency unregulated drugs often carry high risks of poor outcomes such as overdose and death, with the relevant literature cited. This will be more informative for the reader.

**Response:**

We agree that our original phrasing was ambiguous and unnecessarily emotive. What we intended to convey is that fentanyl and benzodiazepines are increasingly found in the unregulated opioid supply in British Columbia, which increases the risk of overdose and complicates overdose response and treatment. To address this, we have revised the text to use more precise, plain language and added explanation regarding the public health risks.

The revised text can be found on page 35 line 460:

*“Likewise, in British Columbia, Canada, drug checking data show that the illicit opioid supply is highly variable, with frequent contamination involving fentanyl. About half of analyzed opioid samples also contained benzodiazepines[109]. The polysubstance nature of the unregulated supply, combined with the high variability of these compounds, increases the risk of overdose and hospital admissions and underscores how unregulated drug markets produce unpredictable, high-potency substances with serious public health consequences (Khadka, 2023).”*

**Comment:**

p. 36 line 560, longer follow-up periods are just one component of temporality that would permit causal inference, I suggest that your recommendation also includes the suggestion that studies are designed in a way that permits causal inferences to be made with respect to the relationship between substance use and mental health conditions. This would include measuring the timing of initiation of substance use and commencement of mental health conditions relative to each other.

**Response:**

Thank you for this important point. We have revised the text to emphasize that future studies should not only extend follow-up periods but also be designed to allow causal inferences, particularly by capturing the sequencing of events such as the timing of substance use initiation and the onset of mental health conditions.

The revised text can be found on page 36 line 493:

*“A key consideration is that study designs should address temporality by incorporating longer follow-up periods, as well as by capturing the sequencing of events, allowing for causal inferences. Current approaches often demonstrate associations without clarifying whether mental health outcomes follow substance use or policy changes. Causal inference would be strengthened if the timing of substance use initiation were clearly measured relative to the onset of mental health conditions.”*
